# Pseudoalteromonas arabiensis sp. nov., a marine polysaccharide-producing bacterium

**DOI:** 10.1099/ijs.0.055459-0

**Published:** 2013-08

**Authors:** Hidetoshi Matsuyama, Hideki Minami, Hirokazu Kasahara, Yoshihisa Kato, Masafumi Murayama, Isao Yumoto

In this paper (doi:10.1099/ijs.0.043604-0), *Pseudoalteromonas arabiensis* sp. nov. is erroneously cited as *Pseudoalteromonas arabianensis* sp. nov. in the title of the species description (page 1807).

doi:10.1099/ijs.0.055459-0

